# Death Anxiety and Loneliness among Older Adults: Role of Parental Self-Efficacy

**DOI:** 10.3390/ijerph18189857

**Published:** 2021-09-18

**Authors:** Lee Greenblatt-Kimron, Miri Kestler-Peleg, Ahuva Even-Zohar, Osnat Lavenda

**Affiliations:** School of Social Work, Ariel University, Ariel 40700, Israel; mirikp@ariel.ac.il (M.K.-P.); ahuvaez@ariel.ac.il (A.E.-Z.); osnatla@ariel.ac.il (O.L.)

**Keywords:** death anxiety, parental self-efficacy, loneliness, older adults

## Abstract

Death anxiety and loneliness are major issues for older people. The present study aimed to broaden the understanding of factors that are linked with increased loneliness in old age by examining the association between death anxiety and loneliness, and the role of an unexplored variable among older adults, namely, parental self-efficacy. A convenience sample of 362 Israeli parents over the age of 65 was recruited through means of social media. Participants completed self-reported questionnaires, which included background characteristics, death anxiety, parental self-efficacy, and loneliness measures. The findings showed that death anxiety was positively associated with loneliness among older adults. The findings also confirmed that parental self-efficacy moderated this association in this population. We concluded that the combination of death anxiety and low parental self-efficacy identified a group of older adults that are at higher risk of developing increased loneliness levels. Mental health professionals should consider intergenerational relationships as a fundamental component of older adults’ daily lives, focusing on parental self-efficacy in old age, as this appears to be a resilience resource.

## 1. Introduction

The parent–child bond plays a vital part in peoples’ well-being across their lifespans [1,2]. Therefore, in investigating factors that are associated with psychological well-being in later life, there is great importance in examining the relationship between parents and their adult children [3]. Furthermore, intimate relations with adult children are known to enhance older adults’ emotional health by increasing self-efficacy perceptions, while negative feelings toward adult children or unsatisfying relationships with them were associated with psychological distress [4,5,6], including loneliness [7]. 

Loneliness is common among older adults [8] and associations were found between changes in relationships that are linked with old age and increased loneliness [9]. Death anxiety is also a significant factor among older people, as death is more plausible at this age, and preparing for one’s death is known in the literature to be an essential developmental task of older adulthood, namely, reaching a sense of integrity versus despair at the end stage of life [10]. Continuity theory [11,12] postulates that older people should engage in activities that maintain their past experiences, behaviors, and relationships. According to this definition, continuity is conceptualized as the consistency of motifs across time and consists of internal continuity (e.g., personal values and perspectives) and external continuity (e.g., social roles and social relationships) [11]. In terms of social roles and relationships, there is an increase in the number of studies that examined parenting aspects among older parents of adults with disabilities [13]; however, there is a need for further research with regard to the parent–child relationship and life satisfaction among older adults in general [14]. Pursuant to these theories and findings, this topic is of importance, as studies have highlighted the continuity of the parental role and its effect on older parents’ identity, well-being, and mental condition [14,15]. Providing instrumental support was noted to be a distinguishing feature of parenting in later life, whereby older adults tend to provide financial support to their adult children [14]. Nevertheless, the authors [14] emphasize the importance of the emotional aspects of the parent–child relationship in old age. Specifically, higher emotional states were linked with the older adults’ ability to provide support to their adult children [16,17], such as higher self-esteem and feelings of independence [14]. It was noted in the literature that high self-esteem is reflected in self-efficacy [18]. In line with the above, the present study aimed to broaden the existing research by examining a new parenting aspect in old age, namely, parental self-efficacy, and its role in the association between death anxiety and loneliness among older adults.

### 1.1. Loneliness in Old Age

Loneliness describes a personal feeling that indicates a person’s perception of the unfulfillment of intimate, emotional, and social needs [19,20]. Henceforth, a definition of loneliness is the diversity between desired and existing social relations [21]. Dominant contributing factors of loneliness are the loss or absence of relationships with others [22] and, in particular, deficiency in the quality of relationships, rather than quantity [23]. Old age, therefore, produces circumstances that result in people feeling lonelier than younger people [24] as a result of the losses associated with old age, including declining physical and mental health and fewer intimate relationships [25] due to the loss of family and friends [26]. Studies indicated that about one-third of older people endure some degree of loneliness at the end of their life [27,28,29], while those aged 80 and over report constant feelings of loneliness [20]. Nevertheless, many older people perceive loneliness to be a natural and predestined part of aging [30]. Various associations were found in studies between loneliness and adverse mental and physical health outcomes in old age (for a review, see [31]). Mental outcomes include reduced subjective well-being and aging satisfaction [32], depression [33,34], anxiety [35], suicidality, and reduced positive emotions [36,37]. Loneliness in older adults was also associated with lower levels of self-rated health [38], concerns regarding mortality [39], accelerated decreases in physiological resilience [22], and reduced cognitive function [40]. Therefore, loneliness has adverse physical and mental implications for the older population, making it essential that the matter be taken seriously [41].

Previous studies found a positive relationship between loneliness and death anxiety [42,43]. Moreover, death anxiety was found to have a causal role in various mental health conditions [44,45,46]. Yet, despite the existing research on death anxiety among older adults [47], the associations linked with this construct are not well understood in old age [48,49]. The present study, therefore, seeks to explore the relationship between these two important constructs, namely, death anxiety and loneliness, among older adults. 

### 1.2. Death Anxiety 

Death is omnipresent and may produce anxiety [50]. Death anxiety involves a multitude of death attitudes that are commonly defined as anxiety that people experience in anticipation of the state in which they no longer exist [51]. Therefore, it describes a person’s anxiety and fear regarding death [52] and is characterized by threat, unease, and discomfort with death [53]. People’s sentience of death in general, and their personal death in particular, was depicted in various psychological and existential theories [54]. According to existential theories, death anxiety is unavoidable anxiety that is experienced before entering the person’s consciousness level [55]. Cognitive theories perceive death anxiety as having a vital role in other anxiety disorders [56]. Studies suggest that people with a positive outlook on life experience less death anxiety [57]. In contrast, despair [58], low perception of social support [59], pessimism among older adults [48], and loneliness [60] were associated with high death anxiety disorders [61]. Moreover, Cohen-Mansfield and Parpura-Gill [62] developed a theoretical model of loneliness (MODEL), which demonstrated that the most important predictor of loneliness is self-efficacy in social situations. 

Terror management theory (TMT), which is a social psychological theory based on existential, psychodynamic, and evolutionary contexts, postulates that people devote mental energy to discount mortality, resulting from the awareness of their inevitable death [63]. According to TMT [64], fears regarding mortality activate two psychological mechanisms, namely, cultural worldviews and self-esteem, which protect people from this awareness. Other researchers [65] expanded this theory by including an additional mechanism: interpersonal relationships. Specifically, preserving close relationships renders a metaphorical buffer that protects people from the fear of death, while shattering close relationships augments the awareness of death [65]. In line with this, it was found that children’s connections to their parents continue to protect them from death anxiety long after leaving the “nest” [66]. However, the role of the older parent has yet to be examined in this light. In addition, as it was recently suggested that family relations and loneliness interact [67], the present study aimed to broaden the existing knowledge regarding constructs and variables that predict loneliness among older people with a focus on a specific aspect of family relations and self-efficacy, namely, parental self-efficacy. To the best of our knowledge, a lacuna exists regarding the study of parental self-efficacy in the older population. Moreover, in line with Erikson’s developmental task of older adulthood (integrity versus despair) [10], feeling anxious about death in old age may decrease the sense of parental self-efficacy in this population. Therefore, based on the above, as well as research that highlights the importance of reciprocity in intergenerational relations between older parents and their adult children [14], in the present study, it was proposed that parental self-efficacy will protect older adults from existential fears about mortality, and in this way, serve as a moderator in the relationship between death anxiety and loneliness in old age. 

### 1.3. Parental Self-Efficacy

Self-efficacy, pursuant to the social-cognitive theory [68], is people’s belief in their ability to execute activities that will produce predetermined outcomes. People with low self-efficacy tend to internalize failure and desist quickly, resulting in feelings of depression, helplessness [68], anxiety, fear, and apprehension [69]. Self-efficacy is assumed to be engaged on a global level, as well as in diverse realms of life [68]. Reduced self-efficacy that is exhibited by older people in social situations may be due to several factors, including self-deprecation resulting from the physical changes that come with age, loss of previous social roles, and lack of practice in initiating or developing new social connections [62]. As interpersonal self-efficacy was found to be a strong predictor of loneliness [70], the current study examined self-efficacy among older adults in one distinct realm in life, specifically, parental self-efficacy.

Parental self-efficacy refers to parents’ perceptions and evaluations of their competence in their performance as parents and their ability to accomplish parenting tasks [71,72]. In contrast, low parental self-efficacy was associated with tiredness [73], anxiety [74,75], and depression [75,76]. Important to the current study, previous research found an association between parents’ loneliness and their parental self-efficacy [75,77]. In particular, parental self-efficacy was found to be negatively associated with loneliness among parents at younger ages. Nevertheless, to the best of our knowledge, previous studies that investigated parental self-efficacy focused on younger populations [75,77], yet did not focus on the older population. 

In summary, the present study aimed to broaden the understanding of factors that are linked with increased loneliness in old age. Based on previous findings that indicate an association between self-efficacy and anxiety [69] and between death anxiety and loneliness [74,75], this study focused on parental self-efficacy as a moderator of the relationship between death anxiety and loneliness among older adults. As parenting is still central and dominant in adulthood, in a way that may even outweigh death anxiety, it may have an impact on loneliness levels. We first hypothesized that higher levels of death anxiety would be related to higher levels of loneliness among older adults. The second hypothesis maintained that parental self-efficacy would moderate this association; in other words, relative to higher levels of parental self-efficacy, older adults with lower levels of parental self-efficacy would exhibit a stronger positive relationship between death anxiety and loneliness. 

## 2. Materials and Methods 

### 2.1. Participants and Procedure

The data was gathered using a convenience sample of 362 Israeli parents above the age of 65 (*MIN* = 65, *MAX* = 91, *M* = 72.1, *SD* = 5.9) who were recruited through means of social media (such as Facebook groups, WhatsApp groups, and internet forums). About two-thirds of the sample (63.7%) were female and most of the sample were married or living with a partner (74.5%). The rest of the sample were almost equally divided between being divorced (12.7%) and widowed (12.2%). Participants reported an average of 14.6 years of education (*SD* = 3.3) and 3.5 children on average (*SD* = 1.5). In terms of living arrangements, about two-thirds of the sample reported living with their partner (66.1%), only a few reported living with extended family (3.6%) or in an assisted living facility (5.0%), and one-fourth reported living on their own (25.3%). Most of the sample reported their economic status as “good” or “very good” (62.7%).

After receiving approval from the Ariel University Institutional Review Board, a link to an electronic form was disseminated through social networks and social media groups, where participants were asked to sign an informed consent form. Participants received no reward for participating in the study. 

### 2.2. Measures

Participants were asked to report information regarding their age, gender, marital status, perception of their economic status (on a Likert-type scale ranging from 1 = “not good at all” to 5 = “very good”), level of education (i.e., number of years of education), number of children, and their living arrangements (1 = “living on my own,” 2 = “living with my partner,” 3 = “living with extended family,” and 4 = “living in an assisted living facility”). 

Parental self-efficacy was assessed using an 18-item scale based on the Parenting Sense of Competence Questionnaire (PSOC) developed by Johnston and Mash [78]. This scale assesses parent’s sense of success in fulfilling the parenting role. To fit the questionnaire to older adults the term “child” was replaced with “son/daughter” in all the relevant items (e.g., being a parent is manageable, and any problems are easily solved). In addition, to verify the validity of the measure to older adults, a CFA was conducted, which revealed a good fit with the data (GFI = 0.91, CFI = 0.86, NFI = 0.81, RMSEA = 0.07). Participants were asked to indicate the degree to which they agreed with the statements on a four-point Likert scale ranging from 1 (not at all) to 4 (very much). In the current study, Cronbach’s alpha was 0.79. 

Death anxiety was assessed using a 12-item scale developed by Carmel and Mutran [79] based on the work of Templer [80], Wong et al. [81], and Thorson and Powell [82]. The scale considers both fear of death (6 items) and fear of dying (6 items) (e.g., I am very afraid of death). Participants were asked to indicate the extent to which they agreed with the statement in each item on a five-point Likert scale ranging from 1 (completely disagree) to 5 (completely agree). In the current study, Cronbach’s alpha was 0.85.

Loneliness was assessed using the short version of the Revised UCLA Loneliness Scale [83] developed by Hughes and his colleagues [84]. This is a 3-item scale that considers the perceived sense of loneliness that is reflected by the feeling of isolation, lack of companionship, and feeling left out (e.g., I feel isolated from others). Responses are given on a three-point Likert scale: 1 (hardly ever), 2 (some of the time), and 3 (often). A higher mean score reflected a higher level of loneliness. Cronbach’s alpha in the present study was 0.83.

### 2.3. Data Analysis

The examination of the moderation model was conducted using the IBM SPSS statistic package (SPSS-26) (IBM SPSS Statistics for Windows, Version 26.0. IBM Corp.: Armonk, NY, USA). First, correlations were examined to establish the preliminary associations between the study variables. Then, a moderation model was tested, using model 4 PROCESS 3.4 macro for SPSS [85], with a bias-corrected bootstrap with 5000 resamples. 

To prevent the bias of results due to demographic variables that were found to be associated with the dependent and independent variables (i.e., age, gender, marital status, socio-economic status, housing arrangements, years of education, and number of children), the analysis was conducted while controlling for these variables. 

## 3. Results

The correlations between the study’s variables are presented in Table 1. The correlations indicated that death anxiety in old age was positively associated with loneliness (*r* = 0.195, *p* < 0.001) and negatively associated with parental self-efficacy (*r* = −0.123, *p* = 0.021). Parental self-efficacy was negatively associated with loneliness (*r* = −0.463, *p* < 0.001). As demographic variables were found to be associated with at least one of the study’s variables, they were held constant during the examination of the moderation effect to prevent a possible bias of the results. 

**Figure 1:**
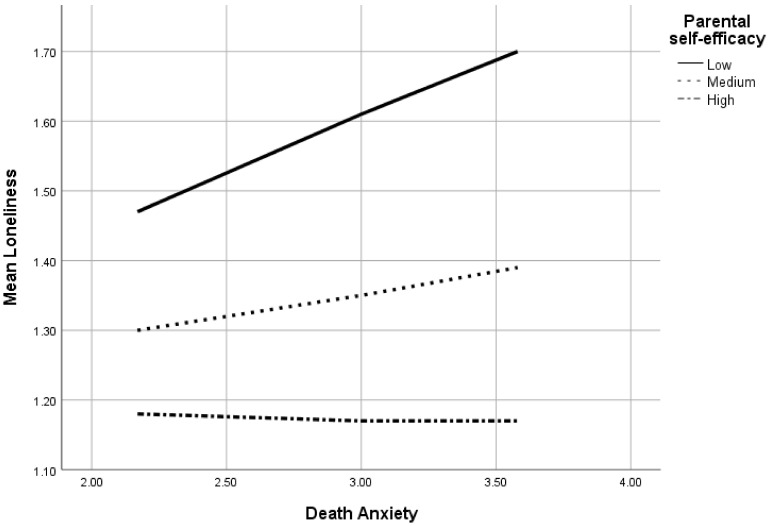
The moderating role of parental self-efficacy in the association between death anxiety and loneliness.

The model explained 29.5% of the variance in loneliness. As indicated in Figure 1, the significant interaction (B = −0.18, CI = [−0.35, −0.02]) was probed with a computational procedure [85] using equations estimating effects when parental self-efficacy values were at ±1 SD from the mean. At an average level and +1 SD (i.e., mean and high level of parental self-efficacy), the effects of death anxiety on loneliness were non-significant (B = 0.06, CI = [−0.01, 0.13] and B = −0.01, CI = [−0.11, 0.09], respectively). However, the lower the level of parental self-efficacy, the stronger the association between death anxiety and loneliness. Namely, at −1 SD (i.e., low level of parental self-efficacy), death anxiety was positively associated with loneliness (B = 0.16, CI = [0.06, 0.26]).

Levels of loneliness were a function of death anxiety among participants with low-, medium- and high-level parental self-efficacies. According to the figure, participants with the lowest levels of self-efficacy reported the highest levels of loneliness, which was increased the more the levels of death anxiety were experienced.

## 4. Discussion

To the best of our knowledge, the present study was the first to examine the relationship between death anxiety and loneliness among older adults and, in particular, the moderating role of parental self-efficacy in this relationship. As hypothesized, older adults who reported higher levels of death anxiety tended to also report higher levels of loneliness. Moreover, parental self-efficacy of older adults was found to play a crucial role as a moderator of this association. The present findings indicated that the association between death anxiety and loneliness was no longer significant when the reported levels of parental self-efficacy were average and above. Nevertheless, among older adults who reported lower levels of parental self-efficacy, this association was strong and significant. 

Consistent with the first study hypothesis, high levels of death anxiety among older adults were found to be related to higher levels of loneliness. This association highlights the contribution of death anxiety to different mental states, as was found in previous studies [44,45,46]. In addition, this association supports the positive relationship between death anxiety and loneliness that was found in previous studies [42,43]. It appears that people’s acknowledgment of the upcoming end of their life journey and the fear such acknowledgment arouses emphasizes the need for and the lack of intimate and significant relationships. Moreover, vice versa, the unfulfilled need for these relationships in older age stresses the impending death, which, in turn, evokes death anxiety. 

Of importance to this study is the finding that older adults who reported lower parental self-efficacy also reported higher levels of loneliness. This finding underscores the effect of parental self-efficacy on loneliness in old age, where older adults with low parental self-efficacy experienced higher levels of loneliness. Although the direction of causality is unknown, the current findings are in line with the negative association found between parents’ loneliness and their parental self-efficacy among younger populations [75,77]. Therefore, it appears that even in old age, as at younger ages, parents’ confidence in their ability to function as a parent is related to their perception of themselves in broader social contexts. 

Supporting the second study hypothesis, which maintained that parental self-efficacy would moderate the relationship between death anxiety and loneliness, it may be assumed that older adults who perceived themselves as having lower parental self-efficacy were more vulnerable to the adverse effects of death anxiety, as reflected by a higher level of loneliness. As the relationship with older children is so significant in the lives of older people, it appears that when an older person feels unable to function as a parent, the effect of death anxiety, which is also pronounced at this age, may intensify the feeling of loneliness. This finding supports previous research that found interpersonal relationships to protect people from the awareness of their inevitable death [65,66]. Therefore, this study highlights the idea that the sense of parental self-efficacy at this age may not only negate the destructive link that exists between death anxiety and loneliness but may also be a resilience resource, as the findings show that when a parent has an average parental capacity and above, the interaction between anxiety and loneliness decreases. Moreover, at an older age, parents suffer losses of meaningful relationships and their yearning for social interactions enhances [86]. Consequently, findings show associations between positive relationships with their adult children and better mental health among older parents [87,88]. Hence, it appears that higher parental self-efficacy in old age may be a potential resource that can contribute to elevating the adverse effects of death anxiety, as well as alleviating feelings of loneliness. 

The present study has several limitations; hence, the findings should be interpreted cautiously. First, it used a cross-sectional design; therefore, causality should be attributed with caution. In particular, it is difficult to determine whether the moderating effect was due to self-efficacy itself or the nature of the parent–child relationship. It is recommended to examine this effect in future studies, with a focus on the characteristics of parent–child relations in old age. Second, the study sample was formed using social networks and may be biased toward older adults exposed to these networks. As a result, there are no data on response rates, which may limit the generalizability of the findings. It is recommended for future studies to strive to collect data from a wide range of populations in terms of gender, age, culture, and data collection methods. Third, the study was based on self-reported questionnaires, which may reflect social desirability and, therefore, limit the ability to generalize the findings. Moreover, the sequence of presented measures in the questionnaire may have biased participants’ responses and, therefore, should be further tested using counterbalancing methods. Despite the above-mentioned limitations, the present study aimed to decrease the gap in the literature concerning the role of parental self-efficacy among older adults and, in particular, its relationship with the association between death anxiety and loneliness in old age. Moreover, this study, as far as we know, was the first to examine the moderating role of parental self-efficacy in the relationship between death anxiety and loneliness in general and, in particular, among older adults. The findings have theoretical and practical implications for improving the quality of life of older adults by focusing on an innovative approach to decrease loneliness in this population.

Theoretically, the findings strengthen earlier results showing that lower parental self-efficacy is a risk factor for mental health. Nevertheless, it should be stressed that a lacuna exists regarding parental self-efficacy in the older population. As these results are preliminary, it is highly recommended that future studies continue to investigate the effect of parental self-efficacy on the mental health of older adults and the directionality of these relationships. Moreover, further research should examine the moderating effect of parental self-efficacy in the relationships between death anxiety and other mental health outcomes in older adults. 

On a practical level, the combination of death anxiety, together with low parental self-efficacy, identifies a group of older adults at higher risk for developing increased levels of loneliness. Mental health professionals should focus on the role of intergenerational relationships, with parenting being considered a fundamental component of older adults’ daily lives to develop older adults’ parental self-efficacy, as this appears to be a resilience resource. Cognitive restructuring or social skills training may improve social self-efficacy, which may be an efficient cognitive-behavioral treatment for loneliness in older adults [62]. Therefore, it may be worthwhile to use parental self-efficacy as a measure of the risk of developing loneliness among older adults. In summary, interventions that promote parental self-efficacy among older adults should be implemented, with the goal of increasing older adults’ personal feeling of capability and self-esteem, while maybe even reducing their levels of loneliness and thereby improving their quality of life. Additionally, mental health professionals should approach the subject of death in the clinical setting, as death anxiety that is experienced at this stage of life may be crucial in understanding the risk factors of loneliness among older people.

## 5. Conclusions

Loneliness has adverse physical and mental implications for the older population; therefore, it is crucial to be cognizant of these effects. Our study showed how the combination of death anxiety and low parental self-efficacy identified a group of older adults at higher risk of developing increased loneliness levels. Mental health professionals should recognize intergenerational relationships as an underlying component of older adults’ daily lives, focusing on parental self-efficacy in old age, as this appears to be a resilience resource. Additionally, future studies should continue to investigate the effect of parental self-efficacy on the mental health of older adults and the directionality of these relationships.

## Figures and Tables

**Table 1:** Correlation coefficients between the study’s variables.

Variable Name	Mean (SD)	1	2	3	4	5	6	7	8	9
1. Death anxiety	2921 (0.714)	1								
2. Parental self-efficacy	3235 (0.440)	−0.123 *	1							
3. Loneliness	1388 (0.493)	0.195 **	−0.463 **	1						
4. Gender	N/A	0.112 *	0.130 *	−0.04	1					
5. Marital status	N/A	0.018	−0.012	0.137 *	0.167 **	1				
6. SES	N/A	−0.278 **	0.257 **	−0.248 **	−0.029	−0.086	1			
7. Housing	N/A	−0.097	−0.114 *	−0.016	−0.112 *	−0.334 **	0.074	1		
8. Years of education	14,560 (3304)	−0.126 *	0.111 *	−0.207 **	−0.017	−0.074	0.205 **	0.085	1	
9. Number of children	3499 (1498)	−0.119 *	0.113 *	−0.046	0.047	−0.021	0.005	−0.093	0.062	1
10. Age	72,130 (5886)	−0.084	−0.019	0.120 *	−0.087	0.278 **	−0.058	0.086	−0.104	0.004

* *p* < 0.05; ** *p* < 0.01.
